# The Mystification of Multifocal IOLs Needs Unfolding

**DOI:** 10.18502/jovr.v19i2.16427

**Published:** 2024-06-21

**Authors:** Mehran Zarei-Ghanavati, Christopher Liu

**Affiliations:** ^1^Farabi Eye Hospital, Tehran University of Medical Sciences, Tehran, Iran; ^2^Tongdean Eye Clinic, UK; ^3^National Treatment Centre, Highland, UK; ^4^Brighton and Sussex Medical School, UK; ^6^Mehran Zarei-Ghanavati: http://orcid.org/0000-0002-0679-8055

The journey from Sir Harold Ridley's implantation of the first intraocular lens in 1949,^[1]^ aimed at achieving distance spectacle-free vision, to Pearce's first bifocal IOL implantation in 1986, to provide near vision independence, shows a significant evolution in cataract surgery.^[2]^

The market for multifocal IOLs is growing, and new multifocal IOLs are being designed every few years. Although the new technology may provide better outcomes, it sometimes may be confusing for clinicians when encountering commercial multifocal labelling X-WAVE ${\;}^{TM}$ Technology for AcrySof IQ VivityⓇ or OptiBlue for TECNIS Synergy. It seems that the label for this technology was chosen to be more mysterious than informative. There is also controversy about the classification and indications for the uses of various types of multifocal IOLs. The monoculture of multifocal IOLs encompasses various options, including bifocal, trifocal, multifocal, extended depth of focus (EDOF), and enhanced monofocal IOLs, among others. Moreover, these IOLs, especially EDOF IOLs, are variably combined with monovision.

Confusing multiple-choice options of multifocal IOLs with inadequate explanations can discourage surgeons from choosing any multifocal IOLs. They might feel they lack scientific mastery in this field. Many surgeons and patients might be awaiting a consensus in this field before opting for multifocal IOLs over monofocal ones. Establishing the first consensus on the classification of multifocal IOLs is a crucial step in advancing this field. While the American National Standards Institute (ANSI) has defined criteria for EDOF and enhanced monofocal IOLs, it has some limitations.^[3]^ There is a suggestion to use the defocus curve for the functional classification of multifocal IOLs.^[4]^

The literature about the characteristics and optical outcomes of multifocal IOLs provides valuable insights. There are reports of lab analysis of the optical quality of different multifocal IOLs, often utilizing the modulation transfer function (MTF) curve to assess their optical performance.^[5,6]^ While this information is valuable, its clinical implications remain uncertain because individual factors like corneal aberration, dynamic pupil size, and visual neuroadaptations are often overlooked.^[7]^

The aforementioned limitations and disputes underscore the importance of a clinical approach to report and demonstrate outcomes of multifocal IOLs using large sample sizes to provide practical and real-world insights for their application. Moreover, these studies should adhere to standardized protocols to ensure consistent reporting of outcomes and incorporate often overlooked metrics, such as the real-world performance of multifocal IOLs in near vision under various lighting conditions, or different materials (digital screen versus book), reading speeds, and so on.

In the current issue of *Journal of Ophthalmic and Vision Research (JOVR)*, a study conducted by Kothari et al aimed to assess the clinical outcomes of four multifocal IOLs: ReSTOR SN6AD1, Tecnis ZKB00, Symfony ZXR00, and PanOptix TFNT00.^[8]^ Although the presence of halos/glare was notable (affecting over 60% of patients), it did not significantly differ among the various IOLs. However, spectacle independence was found to be lower for ReSTOR and PanOptix. The research also revealed that overall self-reported visual function following the implantation of multifocal IOLs is linked to spectacle independence rather than optical phenomena. These real-world clinical evaluations comparing different multifocal IOLs offer valuable perspectives. Despite the use of various generations of multifocal IOLs in this study, quite similar outcomes were achieved, except for a higher percentage of patients implanted with Symfony IOLs needing glasses for near-vision tasks.

In summary, beyond advancements in multifocal IOL technology for improved clinical outcomes, addressing patients' expectations regarding uncorrected near vision is crucial. Amidst the elaborate lab optical assessments of multifocal IOLs and the saturation of technological terminology, clear communication through clinical comparisons of multifocal IOLs may offer a more straightforward message for surgeons. Furthermore, sharing this information with patients may assist them in making informed decisions regarding the choice of a multifocal IOL.
